# Intramammary Ethno-Veterinary Formulation in Bovine Mastitis Treatment for Optimization of Antibiotic Use

**DOI:** 10.3390/pathogens12020259

**Published:** 2023-02-06

**Authors:** Dragana Tomanić, Nebojša Kladar, Miodrag Radinović, Ivan Stančić, Mihajlo Erdeljan, Jovan Stanojević, Ivan Galić, Katarina Bijelić, Zorana Kovačević

**Affiliations:** 1 Department of Veterinary Medicine, Faculty of Agriculture, University of Novi Sad, Trg Dositeja Obradovica 8, 21000 Novi Sad, Serbia; 2 Department of Pharmacy, Faculty of Medicine, University of Novi Sad, Hajduk Veljkova 3, 21000 Novi Sad, Serbia

**Keywords:** essential oils, ethno-veterinary, mastitis, Phyto-Bomat, antimicrobial resistance

## Abstract

Increasing pressure on animal and human health as well as food security, due to antimicrobial resistance, is leading to increased demand for natural-product-derived antibiotics worldwide. Considering the importance of bovine mastitis as well as the huge challenge posed by mastitis therapy in cattle production, the main goal of this research was to evaluate the effectiveness of the essential oil-based pharmaceutical (Phyto-Bomat), as an alternative to the existing treatment with antibiotics. The therapeutic response of the cows was monitored using clinical and bacteriological cure of the proposed formulation. Among 550 dairy cows, 75 were diagnosed with a clinical or subclinical form of mastitis and divided into three experimental groups: treated with antibiotic; Phyto-Bomat; antibiotic and Phyto-Bomat. Indicators of bacteriological cure were somatic cell count (SCC), total bacterial count, and causative agent isolation, while the clinical cure was accessed by observing physical udder conditions. All mentioned indicators were assessed before and after the treatment (1st and 7th day post-treatment), with all of the three therapeutic protocols. After Phyto-Bomat treatment, no visible signs of irritation were noticed, while no significant effect in reducing SCC and total bacterial count was observed. Phyto-Bomat has shown promising results in the treatment of mastitis, without the risk of udder irritation, alone or in combination with antibiotics as part of a mastitis control program during lactation.

## 1. Introduction

Antimicrobial resistance (AMR) has been recognized as a global health problem affecting both human and veterinary medicine, being associated with increased morbidity, mortality and societal costs [1,2]. It is predicted that by 2050, AMR will contribute to 10 million deaths per year, causing $100 trillion economic loss [3], as well as around 11% loss in the production of livestock [4,5]. Antimicrobial misuse in therapy and prophylaxis plays a role in the development and spreading of AMR, especially as many of the antibiotics used in animals are the same, or are in the same class, as antibiotics used in humans [1,6]. Additionally, an emerging concern is the occurrence and spread of AMR in the environment [7] since the manure is commonly used for the fertilization of agricultural soils potentially resulting in a high level of antibiotic accumulation in the environment [8,9].

Tackling AMR is also a top priority for the WHO, which has emphasized the importance of taking a One Health approach with the recognition that animal, human and environmental health are linked in addressing this problem [1]. AMR is an inevitable consequence of antibiotic use in veterinary medicine, leading to the limitation of therapeutic options available nowadays [10]. To address this crises, antimicrobial stewardship (AMS) principles are developed to reduce the misuse and overuse of antibiotics and preserve the effectiveness of available antimicrobial agents [3,11,12]. Importantly, according to Tang, et al. [1], broad interventions that restrict global antibiotic use should be conducted in food-producing animals as these appear to be associated with reductions in AMR since global consumption of antibiotics from food animal production is predicted to rise dramatically by 67% by 2030 [9]. Moreover, global purchase of antibiotics related to livestock production is estimated to be 73–100% higher than the purchases for the human health sector [13].

Mastitis is known globally as one of the main disorders leading to antimicrobial treatment of dairy cattle [2,10]. Approximately 60–70% of the antibiotics used in cattle are for the prevention and treatment of mastitis [14]. In addition, it is highlighted as a major veterinary health problem, affecting not only animal health and welfare but also public health, representing a financial burden on global health systems [10]. In addition, mastitis can be classified as a clinical (CM) or subclinical (SCM) form. CM is characterized by sudden onset, alterations of milk composition and appearance, decreased milk production, and the presence of inflammation signs in infected mammary quarters. Whereas CM is easily detected and treated, this is not the case for the most prevalent form. SCM is hardly recognized on time by most farmers because of no visible signs of udder inflammation [15,16]. Additionally, the global prevalence rates of SCM and CM are 42% and 15%, respectively [14].

Antibiotics are not always effective in mastitis therapy because even when a clinical cure is achieved, bacteriological elimination is rarely better than 60% [17]. In addition, the shortcomings of this therapy can include the increased occurrence of antibiotic resistance, reduced food safety and security, and high costs of therapy, as well as the withdrawal period [14,15]. Antimicrobial treatment optimization is essential to keep these drugs effective, since the development of antibiotics has slowed noticeably in the 21st century. In the last 50 years, a new antibiotic class has not been developed for Gram-negative bacteria [18,19]. In the near future, no new classes of antimicrobial agents are expected to become available in veterinary medicine [20]. Along with antimicrobial drug optimization, development of antimicrobial alternatives presents the core principles of AMS. Consequently, there is continuous pressure on veterinarians to reduce the use of antibiotics in the treatment of food-producing animals, which is leading to development of new agents with innovative chemistry and modes of action [19,21,22].

The plant-based ethno-veterinary herbal medicine is one of the alternatives and most sustainable approaches that incorporate traditional beliefs, medicinal plants, knowledge, skills, methods, and practices in animal health care, production, and breeding [14,23,24]. It provides efficient, inexpensive, accessible, and effective therapies, with none or much less side effects [24,25]. Scientific evidence points to the essential oils (EOs) as a possible alternative in replacement of antibiotics due to their promising therapeutic efficacy with minimal side effects, lack of AMR to these phytochemicals, and reduced drug residues in animal products [10,21,26,27].

Concerns about these issues have risen rapidly, affecting the urgent development of new therapeutic approaches in livestock production. For this purpose, the aim of study was to evaluate novel preparation based on EOs originating from Serbia as a therapeutic candidate in bovine mastitis treatment, with previously proven in vitro efficacy. Evaluation will include clinical and bacteriological cures with proposed phytopharmaceutical (Phyto-Bomat) vs. conventional antimicrobial treatment.

## 2. Materials and Methods

### 2.1. Development and Production of Phyto-Bomat

Based on the review of literature data, several different EOs available on the market of the Republic of Serbia were chosen. In this study, four EOs with highest antimicrobial potential against mastitis associated pathogens were selected for intramammary pharmaceutical formulation development, such as the common thyme (*Thymus vulgaris* L.), the wild thyme (*Thymus serpyllum* L.), the oregano (*Origanum vulgare* L.), and the mountain savory (*Satureja montana* L.). The chemical composition and antimicrobial activity of common and wild thyme [28], as well as oregano and mountain savory EOs [29] against bovine mastitis-associated pathogens were previously studied. Moreover, oregano, common and wild thyme EOs were tested on *Serratia marcescens* and *Proteus mirabilis* mastitis associated isolates [30]. The obtained EOs pharmaceutical formulation was further mixed with common marigold (*Calendula officinalis* L.), St. John’s wort (*Hypericum perforatum* L.), and oil macerates (herbal drug:sunflower oil, 1:5) in 15 mL of the formulation, which represents the volume of the one injector.

After the optimization of the concentration of ingredients, a pharmaceutical formulation (Phyto-Bomat) for intramammary use was prepared. The concentration of EOs in the proposed formulation was determined based on the EOs minimal bactericidal concentration (MBC). Moreover, the used MBC values were increased to a certain number of times from the obtained in vitro values to achieve the pharmacological effectiveness of the proposed formulation in the clinical setting. The preparation of the Phyto-Bomat was carried out in the Laboratory of Pharmacology and Toxicology at the Department of Veterinary Medicine, Faculty of Agriculture, University of Novi Sad. The antimicrobial activity of selected EOs was previously tested at the Laboratory of the Scientific Institute for Food Technologies (FINS), University of Novi Sad. In the next step, chemical composition and antimicrobial activity of final pharmaceutical formulation (Phyto-Bomat) were tested [31].

The effectiveness of the final formulation on microorganisms was determined according to the Clinical Laboratory Standards [32], described by Kovacevic, et al. [31]. The minimum inhibitory concentration (MIC) was determined as the lowest concentration of the final preparation that prevented the transition of oxidated form to the reduced form of resazurin and was determined by cultivating 100 µL of solution from each well of the microtiter plate in Mueller–Hinton agar (MHA, HiMedia) [33]. MBC was defined as the lowest concentration of the final preparation solution at which 99.9% of inoculated bacteria were killed. The EO-based formulation exhibited antimicrobial activity against the tested mastitis-associated bacteria. The MIC of the formulation for the tested bacterial species ranged from 22.72 mg/mL to 45.4 mg/mL, while the lowest MIC values were found for *E. coli*, *Streptococcus* spp., and *Staphylococcus* spp. strains. The MBCs determined for the EO-based formulation ranged from 45.4 mg/mL to 90.09 mg/mL [31].

### 2.2. Treatment Protocol

The experimental protocol was approved by the Animal Ethics Committee of the Ministry of Agriculture, Forestry and Water Management-Veterinary Directorate (9000-689/2, 6 July 2020). The study was conducted on a dairy farm located in Vojvodina, Republic of Serbia, with 550 Holstein-Friesian cows. All animals included in the clinical trial were 2 to 5 years old, in the different stages of lactation phase, with diagnosed mastitis, without other health problems, except mastitis. Clinical mastitis was diagnosed by clinical examination, while subclinical mastitis was diagnosed by analyzing milk samples. Before the treatment with Phyto-Bomat started, the local tolerance test was performed on cows free of any apparent infection (except mastitis), on the same farm where the clinical trial was performed according to the planned treatment methods. Regarding the local tolerance of intramammary preparations in cows, the guidance according to EMA was used [34]. The evaluation criterion was the cell count in the treated udder quarters in combination with clinical criteria (appearance of the udder and of the milk) and zootechnical criteria (milk yield). Positive results for mastitis were obtained on 75 cows. All animals were divided into three experimental groups, 25 animals per group. The first group of cows was treated with conventional antibiotic treatment, the second group with Phyto-Bomat, and the third group was treated with the combination (Phyto-Bomat and antibiotic). For conventional antibiotic treatment, cows were treated with commercial antibiotic cephalexin (Rilexine^®^), following the manufacturer’s recommendations, after antibiotic susceptibility test was performed according to the treatment protocol on the farm. Phyto-Bomat was applied intramammary twice a day, after morning and evening milking, for 5 consecutive days. Cows treated with the combination of Phyto-Bomat and antibiotic were firstly treated with cephalexin (Rilexine^®^), used intramuscularly, following the manufacturer’s recommendations, then with Phyto-Bomat used twice a day, for 5 consecutive days.

Milk samples were collected before the therapy, one day and seven days after the end of the therapy. Before sampling, the udder and teats were cleaned with water and teats were disinfected and dried. Then, the tip of each teat was disinfected with 70% ethanol. The first streams of milk were discarded, after which 10 milliliters of milk were stored into sterile tubes marked with an ID number of the cow [31]. After the milk samples were collected, they were immediately transported to the Laboratory for testing biological material, food, and animal feed “In vitro LAB” DOO Šabac, Republic of Serbia, under the cold chain (4 °C) for bacteriological testing.

### 2.3. Clinical Cure Rate of Phyto-Bomat

Clinical cure rate was evaluated by performing physical examination (adspection and palpation) of the udder before the treatment, 1 day and 7 days after the finished treatment. The following parameters were monitored: swelling and redness of the udder, presence of clots and flakes in milk, and change of milk color. Furthermore, to evaluate these parameters, the scale for grading physical changes in the udders was created (0 = normal, 1 = mild, 2 = moderate, and 3 = severe) (Supplementary Table S1).

### 2.4. Bacteriological Cure Rate of Phyto-Bomat

Bacteriological cure rate was evaluated on the basis of the pathogen isolation [28], the total bacterial count [35], and the somatic cell count [36]. All evaluations were carried out pre-treatment and post-treatment (1 and 7 days after the finished treatment).

#### 2.4.1. Isolation and Identification of the Causative Agents

Bacterial isolation from milk samples was carried out using microbiological procedures to diagnose mammary gland infection as published by the National Mastitis Council [37]. Milk samples were inoculated with calibrated platinum loop (0.01 mL) onto 2% sheep blood agar (SBA) (Oxoid, Basingstoke, UK) and incubated for 24–48 h at 37 °C in aerobic conditions. Grown microorganisms were examined for their morphological characteristics with Gram staining (Gram-positive or Gram-negative) and further sub cultivated onto selective media: Mannitol Salt Agar (MSA) for staphylococci, Salmonella Shigella Agar (SS) and MacConkey Agar (MC) for Gram-negative enteric pathogens, Edwards agar for streptococci and Tellurite Blood Agar (TBA) for *Corynebacterium* spp. (all obtained from Oxoid, Basingstoke, UK). Plates were incubated aerobically at 37 °C for 24–48 h, and identification of microorganisms was performed based on their biochemical and cultural traits (colony morphology, pigmentation, and hemolytic pattern).

For distinguishing Gram-positive cocci, the catalase test, mannitol fermentation test on MSA, coagulase test, hemolytic pattern (α and β hemolysis), and colony morphology were used. Isolates of *Staphylococcus* spp. and *Micrococcus* spp. were confirmed by identification system for staphylococci and micrococci Analytical Profile Index API Staph tests (API, bioMeraux, Craponne, France). Edwards agar and hydrolysis of esculin were used for streptococci determination [28], and API 20 Strep was used for confirmation of their identification (API, bioMeraux, Craponne, France).

Gram-negative enteric pathogens (*Escherichia coli*, *Proteus mirabilis*, *Serratia marcescens*, and *Klebsiella* spp.) were identified by colony morphology (swarming for *Proteus* spp., red pigment production for *Serratia* spp., and mucoid colonies for *Klebsiella* spp.) and biochemical tests: oxidase activity (positive), lactose fermentation on MC (pink lactose-positive colonies with bile salt precipitation characteristic for *E. coli*), glucose, sucrose, mannose, rhamnose and arabinose fermentation with/without gas production, IMViC test (indole production, methyl-red test, Voges–Proskauer reaction, and citrate utilization), urease test, and hydrogen sulfide production. In addition, each strain was confirmed by API-20 tests (API, bioMeraux, Craponne, France).

*Corynebacterium* spp. was isolated on SBA and TBA and identified by typical microscopic appearance (rod/club-shaped Gram-positive bacilli), colony morphology on SBA (weak β hemolysis) and TBA (gray to black colonies due to potassium tellurite reduction into black tellurium that precipitates on surface of colonies), and biochemical tests (catalase positive, oxidase negative, H_2_S production, methyl-red positive, salicin positive, nitrate reduction and fermentation of glucose, dextrose, arabinose, galactose, fructose, maltose, mannose, and rhamnose).

#### 2.4.2. Total Bacterial Count and Somatic Cell Count

Total bacterial count was estimated according to Institute for Standardization of Serbia (SRPS ISO 4833-1:2014) [35] using the horizontal method for the enumeration of microorganisms—Part 1. Total bacterial count was determined on plate count agar with incubation at 30 ± 1 °C for 72 ± 3. Colonies were counted after specified period of incubation as colony forming units (CFU). Results were expressed in CFU/mL of milk. Examination of somatic cell count (SCC) in milk samples was performed using the microscopic reference method according to the Institute for Standardization of Serbia (SRPS EN ISO 13366-1:2010) [36]. SCC was determined by spreading 0.01 mL of mixed milk from each sample over a 1 cm^2^ area on a glass slide. The slides were left to air-dry and then stained by Newman-Lampert stain and examined microscopically. A result up to 200,000 cells/mL was taken to be a low SCC, since this is generally considered healthy mammary gland [38]. A high SCC was >200,000 cells/mL, as this is a threshold for the presence of intramammary infection [39].

### 2.5. Statistical Analysis

The obtained results were summarized using MS Office Excel (v2016) and statistically processed with Tibco Statistica v13.5. All of the variables were characterized by application of descriptive statistics and further analyzed by means of univariate statistical methods (Kruskal-Wallis test or ANOVA for repeated measures, depending on the type of data) or multivariate statistics (multivariate correspondent analysis). The results were considered significant if *p* < 0.05.

## 3. Results

### 3.1. Clinical Cure Rate of Phyto-Bomat

**Figure 1:**
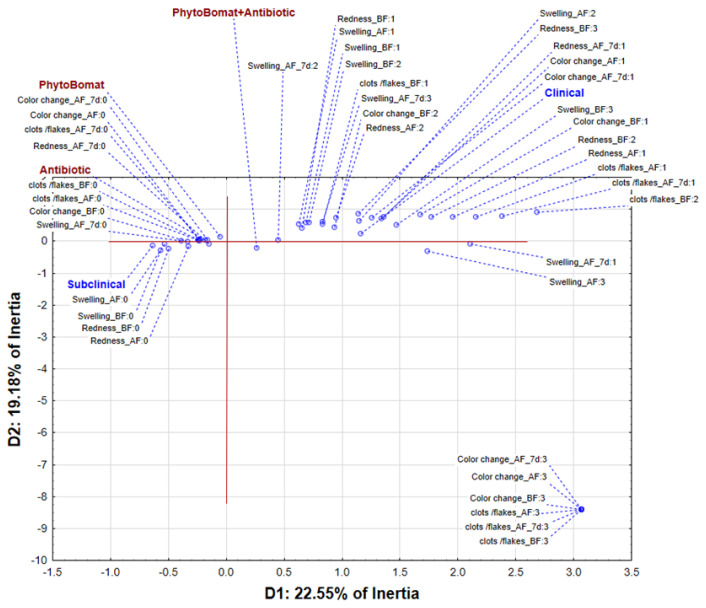
The position of evaluated symptoms scores and applied treatment approach in the space defined by the first two correspondent axes.

The application of multivariate correspondent analysis on dataset describing symptoms of mastitis and their change in relation to type of mastitis and applied treatment shows that the first two dimensions (Ds) describe more than 40% of samples’ inertia (Figure 1). The position of the obtained results in the space defined by the first two correspondent axes shows grouping of samples with subclinical mastitis in the negative part of D1. Although these samples were not characterized by any symptoms at the moment of diagnosis, their treatment with Phyto-Bomat or antibiotic was effective since they did not develop to the clinical mastitis. Generally, it can be noticed that treatment with Phyto-Bomat or antibiotics is associated by relief of the mastitis symptoms. On the other hand, the positive part of D2 is associated with the existence of symptoms in the case of the clinical mastitis; however, it must be stated that they are being heavily reduced in most cases.

### 3.2. Bacteriological Cure Rate of Phyto-Bomat

#### 3.2.1. Bacterial Isolation

**Figure 2:**
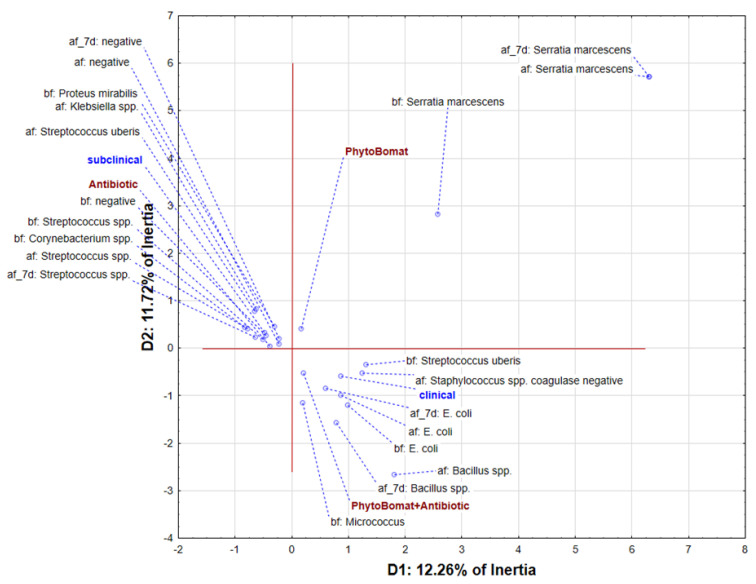
The position of identified bacterial strains and applied treatments in the space defined by the first two correspondence axes.

The application of multivariate correspondent analysis on dataset describing the presence of bacteria before the treatment, after the treatment and seven days after the treatment, as well as diagnosed form of mastitis and applied treatment shows that the first two dimensions describe around 24% of samples’ variability (Figure 2). The position of the analyzed variables shows grouping of samples with subclinical mastitis treated with antibiotics in the negative part of the first correspondent axis (D1). These samples were characterized by isolation of *P. mirabilis*, *Streptococcus* spp., and *Corynebacterium* spp. before the treatment. After the treatment, the results were negative or indicated the presence of *Klebsiella* spp. or *Streptococcus uberis*. Interestingly, it seems that the *Streptococcus* spp. was resistant to treatment by antibiotic. Furthermore, in the positive part of D1 and negative part of the second correspondent axis (D2), samples described as clinical mastitis treated by combination of Phyto-Bomat and antibiotic are located. These samples were characterized by isolation of *S. uberis*, *E. coli* and *Micrococcus* before the treatment, whereas it seems that the treatment was ineffective against *E. coli*. The positive part of D1 and D2 was reserved for *S. marcescens*, which seems to be resistant to any of the applied treatment.

#### 3.2.2. Total Bacterial Count

**Figure 3:**
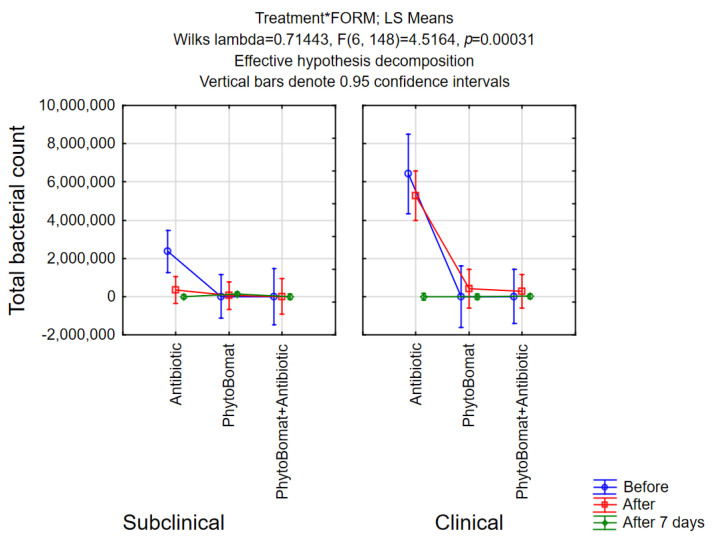
Total bacteria count in milk samples.

Difference between the total bacterial count in the milk samples before, after and 7 days after the treatment in relation to the treatment and the form of mastitis was observed only in the experimental group of cows treated with antibiotics. However, in case of the clinical form of the mastitis, slight increase in evaluated values is recorded after Phyto-Bomat treatment, as well as after the treatment with both Phyto-Bomat and antibiotics being reduced to the level before therapy on 7th day post-treatment (Figure 3).

#### 3.2.3. Somatic Cell Count

**Figure 4:**
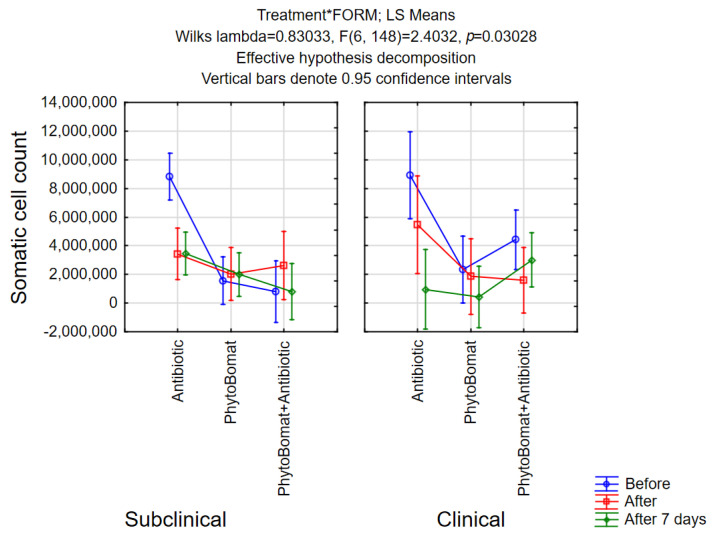
Somatic cell count in milk samples.

Difference between the SCC in the milk samples before, 1 day after and 7 days after the treatment in relation to the treatment and form of mastitis was only observed in experimental group of cows treated with antibiotics. Treatment with antibiotic resulted in SCC reduction post-treatment. However, clinical mastitis resulted in reduction of SCC in the group treated with Phyto-Bomat and the group treated with both Phyto-Bomat and antibiotics 7 days after the treatment (Figure 4).

## 4. Discussion

Although different mastitis control programs exist for many years, they did not provide an adequate solution; therefore, the problem of occurrence of this disease is still present. Even though the use of antibiotics remains as the main treatment strategy, due to the limited effectiveness, herbal approaches are becoming more popular [15]. The possibility of using plant-based pharmaceuticals in controlling bovine mastitis has shown potential in in vitro studies [22,40,41,42,43,44]. In addition, there are only few studies regarding in vivo testing of the efficacy of the natural based formulations in the bovine mastitis treatment [21,45,46,47]. Moreover, only few of them were focused on EO-based products in the bovine mastitis [48,49,50], while Aleksih, et al. [51] and McPhee, et al. [52] were testing Eos in sheep and goats.

Considering the advantages of intramammary application, such as high concentrations of the substance achieved in the milk and low consumption of the antibiotics as the drug is directly infused into the diseased quarter [53], the present study aimed to develop EO-based pharmaceutical formulation (Phyto-Bomat) for intramammary application. As Phyto-Bomat components being generally recognized as safe [54], proposed Eos have been previously tested in vitro (antimicrobial potential, chemical composition, and antioxidant potential) [28,29,30]. The promising results obtained in these studies were applied to the development of pharmaceutical formulation.

Different results of clinical and bacteriological cure for various mastitis treatments have been reported in different studies. Differences in causative agents, mastitis severity, time for cure evaluation, as well as differences in the product formulations and the therapeutic protocol make it difficult to compare studies that evaluated treatment in cows affected with mastitis. Additionally, different pharmaceutical formulation (ointment, solution, gels, sprays, and creams) made with plants could be used for treatment, which can also affect an outcome of therapy. Interestingly, the results in our study have shown that antibiotic and Phyto-Bomat therapy have had similar clinical results, as shown in occurrence of signs of inflammation in both treatments. Furthermore, in case of subclinical form of the mastitis, treatments apparently prevented the development of clinical form, while symptoms were mostly reduced in clinical form [55,56]. Interestingly, it is reported that treatment with PhytoMast^®^ did not have a significant effect on the clinical mastitis resolution on the 4th day, but resulted in a faster clinical recovery [50]. Subclinical mastitis during lactation is not commonly treated with antibiotics because of high treatment costs and generally poor efficacy [53,57]. However, it is shown that antimicrobial treatment of subclinical mastitis form reduced incidence of clinical mastitis [57], which is in accordance with our results and potentially reduces forward transmission compared with leaving quarters untreated. Considering that our study found no difference in clinical cure between mastitis cases treated with antibiotic and Phyto-Bomat, EO-based pharmaceutical formulation proposed in this research could be recommended as alternative for bovine mastitis therapy.

When evaluating alternatives to antibiotics, especially with EO-based formulations, it is important to consider that mammary gland is very susceptible to irritation [39]. Part of Phyto-Bomat local tolerance testing conducted within our study was in line with EMA guideline [34]. Actually, there were no local or systemic side effects observed in any of the cows treated with Phyto-Bomat, which is in agreement with other studies [46,48]. Moreover, improvement in udder conditions was reported by Cho, et al. [46] who used oregano Eos as the therapeutic candidate for clinical bovine mastitis caused by *Staphylococcus aureus* and/or *Escherichia coli*. Furthermore, interesting results were obtained in the studies in which two EO-based commercial products (Cinnatube^®^ and PhytoMast^®^) were tested in cows with mastitis. Actually, both were used intramammary, one for dry cow treatment (Cinnatube^®^) [48] and the other one for lactating cows (PhytoMast^®^) [50]. In these studies, no visible signs of udder irritation and good tolerance after the treatment with those two formulations were reported.

Bacteriological cure after treatment with Phyto-Bomat showed negative results, considering isolation of causative agents after the treatment compared with those before the EO treatment. In addition, some mastitis-associated pathogens (*E. coli* and *S. marcescens*) were still present post-treatment. Based on the current literature, antibiotic therapy has been reported to have little or no effect in treating clinical or subclinical mastitis caused by *E. coli* [58]. Low rate response to therapy can be due to the growing number of recurrent and persistent mastitis cases often caused by the same *E. coli* isolates [58]. Moreover, *S. marcescens* seems to be resistant to any of the applied treatments, which is in accordance with data reporting poor response to antimicrobial therapy with cure rate less than 14% [30]. In addition, *E. coli* and *S. marcescens* are gram-negative bacteria, which are known to be more resistant to Eos than gram-positive bacteria [59]. The lower susceptibility of gram-negative bacteria is explained by the difference in cell wall structure that limits the diffusion of hydrophobic compounds through the lipopolysaccharide envelope [60]. Contrary to our results, in an in vivo study, the treatment conducted by using oregano EO was able to inhibit *E. coli* post-treatment [46].

In the evaluation of bacteriological cure, SCCs have been used as an indicator of the bovine mastitis in dairy cows to estimate the therapeutic effects on the mastitis treatment with different therapeutic approaches. The efficacy of antibiotic therapy in reducing SCC in the milk was higher compared with Phyto-Bomat-only treatment or combination of those two. According to McDougall, et al. [57], antimicrobial treatment of subclinical mastitis resulted in reduced SCC, which is in agreement with results obtained in our study. In Phyto-Bomat treated cows, no significant difference was observed regarding SCC after therapy finished, which is in agreement with Pinedo, et al. [50], indicating no significant differences in the levels of somatic cells after recovery. Contrary to this, Hase, et al. [49] reported significant reduction of SCC on the 5th day of treatment, where significant difference between Mastilep^®^ gel and herbal spray was observed. Cho, et al. [46] also reported significant decrease in SCC post-treatment with oregano EO ointment.

Total bacterial count was used in our study as one more indicator for bacteriological cure. Yang, et al. [45] reported decreased values of total bacterial count after intramammary infusion of *Angelica dahurica* and *Rheum officinale* extracts. Furthermore, intramammary application of 10% solution of thymus and lavender EOs and of the thymus lavender mixture resulted in drastic decrease in the *Staphylococcus* and *Streptococcus* colony count, while external application exhibited even stronger activity reported by Abboud, et al. [54]. Our research results show that the total bacterial count significantly decreased after the antibiotic treatment, while EO treatment with Phyto-Bomat showed no significant difference. On the other hand, in case of the clinical form of the mastitis, slight increase in evaluated values is recorded after Phyto-Bomat treatment, as well as after the treatment with both Phyto-Bomat and antibiotics being reduced to the level before therapy on the 7th day post-treatment. Regarding the use of Phyto-Bomat along with antibiotic therapy, our research results are in agreement with Yap, et al. [61] who stated that the combination of EOs and antibiotics could be employed as a treatment strategy to reduce antibiotic usage, to decrease the adverse effects, and to potentially prevent occurrence of antibiotic resistance.

Finally, contrary to our findings, recent reviews have concluded that it was impossible to make any conclusions on therapeutic efficiency of alternative veterinary products for the treatment of clinical mastitis, due to limited data [62,63]. Thus, our results provide valuable insight in terms of bacteriological cure rate efficiency in the case of clinical and subclinical mastitis.

## 5. Conclusions

When evaluating success of Phyto-Bomat therapy, resolution of symptoms post-treatment, as well as prevention of development clinical mastitis in cases with subclinical mastitis indicate the possibility of its application as a component in mastitis control programs. Promising results obtained in this study provide an excellent base for the extension of the research and application to the dairy industry. Further in vivo studies are recommended to evaluate the potential use in preventive applications, either as an independent or as a supportive therapy. Additionally, it will be interesting to evaluate the cost and effectiveness of Phyto-Bomat vs. conventional antimicrobial mastitis treatment using pharmacoeconomic analysis. Hence, our future work will be focused on the implementation of the pharmacoeconomic analysis in veterinary medicine. Indeed, this powerful tool could help to calculate economic benefits after using this alternative treatment, especially in treatment of subclinical mastitis form since it contributes to most of the financial losses.

## Data Availability

The data used to support the findings of this study are available in the present manuscript.

## Supplementary Materials

The following supporting information can be downloaded at: https://www.mdpi.com/article/10.3390/pathogens12020259/s1, Table S1: Clinical cure rate responses.
