# Supplementary material for: Intramammary Ethno-Veterinary Formulation in Bovine Mastitis Treatment for Optimization of Antibiotic Use
Source: Pathogens. 2023 Feb 6;12(2):259. doi: 10.3390/pathogens12020259 (PMC9962475; doi:10.3390/pathogens12020259)
Supplement: Supplementary file 1 [file pathogens-12-00259-s001.zip › pathogens-2063049-supplementary.pdf]

Table S1: Clinical cure rate responses.

Treatment	Swelling_BEFORE	Swelling_AFTER	Swelling_AFTER_7days	Redness_BEFORE	Redness_AFTER	Redness_AFTER_7days	clots /flakes_BEFORE	clots /flakes_AFTER	clots /flakes_AFTER_7days	Color change_BEFORE	Color change_AFTER	olor change_AFTER_7da	FORM
Antibiotic	0	0	0	0	0	0	0	0	0	0	0	0	subclinical
Antibiotic	2	1	0	1	0	0	1	0	0	0	0	0	clinical
Antibiotic	0	0	0	0	0	0	0	0	0	0	0	0	subclinical
Antibiotic	0	0	0	0	0	0	0	0	0	0	0	0	subclinical
Antibiotic	0	0	0	0	0	0	0	0	0	0	0	0	subclinical
Antibiotic	0	0	0	0	0	0	0	0	0	0	0	0	subclinical
Antibiotic	3	3	1	2	1	0	2	1	1	1	1	1	clinical
Antibiotic	0	0	0	0	0	0	0	0	0	0	0	0	subclinical
Antibiotic	2	1	0	1	0	0	1	0	0	0	0	0	clinical
Antibiotic	0	0	0	0	0	0	0	0	0	0	0	0	subclinical
Antibiotic	0	0	0	0	0	0	0	0	0	0	0	0	subclinical
Antibiotic	0	0	0	0	0	0	0	0	0	0	0	0	subclinical
Antibiotic	0	0	0	0	0	0	0	0	0	0	0	0	subclinical
Antibiotic	3	3	1	2	1	0	2	1	1	1	0	0	clinical
Antibiotic	0	0	0	0	0	0	0	0	0	0	0	0	subclinical
Antibiotic	0	0	0	0	0	0	0	0	0	0	0	0	subclinical
Antibiotic	0	0	0	0	0	0	0	0	0	0	0	0	subclinical
Antibiotic	2	1	0	1	0	0	1	0	0	0	0	0	clinical
Antibiotic	0	0	0	0	0	0	0	0	0	0	0	0	subclinical
Antibiotic	0	0	0	0	0	0	0	0	0	0	0	0	subclinical
Antibiotic	0	0	0	0	0	0	0	0	0	0	0	0	subclinical
Antibiotic	0	0	0	0	0	0	0	0	0	0	0	0	subclinical
Antibiotic	3	3	1	2	1	0	2	1	1	1	0	0	clinical
Antibiotic	0	0	0	0	0	0	0	0	0	0	0	0	subclinical
Antibiotic	0	0	0	0	0	0	0	0	0	0	0	0	subclinical
PhytoBomat	0	0	0	0	0	0	0	0	0	0	0	0	subclinical
PhytoBomat	2	2	3	1	0	0	1	1	0	0	0	0	clinical
PhytoBomat	0	0	0	0	0	0	0	0	0	0	0	0	subclinical
PhytoBomat	3	2	1	1	0	0	0	0	0	1	1	0	clinical
PhytoBomat	3	2	0	1	1	0	0	0	0	0	0	0	clinical
PhytoBomat	0	0	0	0	0	0	0	0	0	0	0	0	subclinical
PhytoBomat	0	0	0	0	0	0	0	0	0	0	0	0	subclinical
PhytoBomat	0	0	0	0	0	0	0	0	0	0	0	0	subclinical
PhytoBomat	3	3	1	1	0	0	1	1	1	2	1	0	clinical
PhytoBomat	0	0	0	0	0	0	0	0	0	0	0	0	subclinical
PhytoBomat	0	0	0	0	0	0	0	0	0	0	0	0	subclinical
PhytoBomat	3	1	1	2	1	0	0	0	0	1	1	0	clinical
PhytoBomat	0	0	0	0	0	0	0	0	0	0	0	0	subclinical
PhytoBomat	0	0	0	0	0	0	0	0	0	0	0	0	subclinical
PhytoBomat	0	0	0	0	0	0	0	0	0	0	0	0	subclinical
PhytoBomat	2	1	0	1	0	0	0	0	0	0	0	0	clinical
PhytoBomat	0	0	0	0	0	0	0	0	0	0	0	0	subclinical
PhytoBomat	0	0	0	0	0	0	0	0	0	0	0	0	subclinical
PhytoBomat	0	0	0	0	0	0	0	0	0	0	0	0	subclinical
PhytoBomat	2	2	1	2	1	1	0	0	0	1	1	1	clinical
PhytoBomat	2	1	0	1	0	0	0	0	0	1	1	1	clinical
PhytoBomat	0	0	0	0	0	0	0	0	0	0	0	0	subclinical
PhytoBomat	0	0	0	0	0	0	0	0	0	0	0	0	subclinical
PhytoBomat	0	0	0	0	0	0	0	0	0	0	0	0	subclinical
PhytoBomat	0	0	0	0	0	0	0	0	0	0	0	0	subclinical
PhytoBomat+Antibiotic	0	3	1	0	0	0	3	3	3	3	3	3	clinical
PhytoBomat+Antibiotic	0	0	0	0	0	0	0	0	0	0	0	0	subclinical
PhytoBomat+Antibiotic	0	0	0	0	0	0	0	0	0	0	0	0	subclinical
PhytoBomat+Antibiotic	0	0	0	0	0	0	0	0	0	0	0	0	subclinical
PhytoBomat+Antibiotic	0	0	0	0	0	0	0	0	0	0	0	0	subclinical
PhytoBomat+Antibiotic	0	0	0	0	0	0	0	0	0	0	0	0	subclinical
PhytoBomat+Antibiotic	0	0	0	0	0	0	0	0	0	0	0	0	subclinical
PhytoBomat+Antibiotic	0	0	0	0	0	0	0	0	0	0	0	0	subclinical
PhytoBomat+Antibiotic	0	0	0	0	0	0	0	0	0	0	0	0	subclinical
PhytoBomat+Antibiotic	0	0	0	0	0	0	0	0	0	0	0	0	subclinical
PhytoBomat+Antibiotic	3	3	2	0	0	0	0	0	0	0	0	0	clinical
PhytoBomat+Antibiotic	3	3	2	1	0	0	0	0	0	0	0	0	clinical
PhytoBomat+Antibiotic	1	1	0	2	2	1	0	0	0	0	0	0	clinical
PhytoBomat+Antibiotic	2	0	0	3	2	1	0	0	0	1	1	1	clinical
PhytoBomat+Antibiotic	2	0	0	0	0	0	0	0	0	2	1	1	clinical
PhytoBomat+Antibiotic	3	3	1	2	1	1	0	1	1	0	0	0	clinical
PhytoBomat+Antibiotic	0	0	0	0	0	0	0	0	0	0	0	0	subclinical
PhytoBomat+Antibiotic	0	0	0	0	0	0	0	0	0	0	0	0	subclinical
PhytoBomat+Antibiotic	3	3	2	0	0	0	0	0	0	0	0	0	clinical
PhytoBomat+Antibiotic	3	3	2	1	0	0	0	0	0	0	0	0	clinical
PhytoBomat+Antibiotic	1	1	0	2	2	1	0	0	0	0	0	0	clinical
PhytoBomat+Antibiotic	2	0	0	3	2	1	0	0	0	1	1	1	clinical
PhytoBomat+Antibiotic	2	0	0	0	0	0	0	0	0	2	1	1	clinical
PhytoBomat+Antibiotic	3	3	1	2	1	1	0	1	1	0	0	0	clinical
PhytoBomat+Antibiotic	0	0	0	0	0	0	0	0	0	0	0	0	subclinical
